# Demand- and supply-side barriers and facilitators of the weekly iron and folic acid supplementation programme in Sidama Region, Ethiopia

**DOI:** 10.1017/jns.2025.10055

**Published:** 2025-11-28

**Authors:** Amelo Bolka, Tafese Bosha, Samson Gebremedhin

**Affiliations:** 1 School of Nutrition, Food Science and Technology, Hawassa University, Hawassa, Ethiopia; 2 School of Public Health, Addis Ababa University, Addis Ababa, Ethiopia

**Keywords:** Barriers, Ethiopia, Facilitators, Sidama Region, Weekly iron and folic acid supplementation

## Abstract

The weekly iron and folic acid supplementation (WIFAS) programme is a school-based initiative designed to reduce iron and folate deficiency anaemia among adolescent girls. In Ethiopia, donor-supported pilot programmes are implementing WIFAS in schools, but evaluations of its demand- and supply-side barriers and facilitators remain limited. This study aimed to explore these barriers and facilitators in the Sidama Region of Ethiopia. An exploratory qualitative study collected data from ten WIFAS-targeted schools using key informant interviews (KIIs) and focus group discussions (FGDs). Participants included purposively selected school directors, WIFAS-trained teachers, health centre heads, district health and education office nutrition focal points, and regional health and education bureau delegates. Ten FGDs were conducted with adolescent girls and their mothers. Thematic analysis was performed using Open Code software to identify emerging themes. This study identified low awareness of iron and folic acid (IFA) tablets, negative community perceptions, fear of side effects, supplementation interruptions due to school closures, and inadequate coverage as key barriers. Structural barriers included poor programme coordination, weak supply chain management, and water scarcity. Facilitators included free IFA tablet distribution, health extension workers’ involvement in awareness campaigns, positive testimonials, local leader support, training, and regular supervision. The WIFAS programme implemented in schools of the Sidama Region faces programmatic and structural barriers. However, facilitators like free IFA distribution, health extension worker involvement, and community support offer opportunities for improvement. These findings highlight the need for addressing barriers while leveraging existing facilitators for enhancing programme success.

## Background

Anaemia is a major global health problem, significantly affecting adolescent girls.^(1)^ Globally, anaemia prevalence varies by country, affecting 33.7% of reproductive-age women. The highest burden is in low- and lower-middle-income countries, especially rural poor households, with western sub-Saharan Africa (47·4%) and south Asia (43·0%) being the most impacted regions.^(2)^ Ethiopia bears a significant burden of anaemia, with a 24% prevalence among reproductive-age women, as reported in the 2016 Ethiopian Demographic Health Survey.^(3)^ Anaemia prevalence among Ethiopian adolescent girls varies widely (23–32%), indicating it is a moderate public health problem.^(4–6)^

Anaemia in reproductive-age women and adolescent girls stems from multiple factors. The primary cause of anaemia in reproductive-age women is iron deficiency, resulting from menstrual losses and diets low in bioavailable iron.^(2)^ Malaria, helminthic infections, and chronic diseases heighten this risk.^(7)^ In developing countries including Ethiopia, factors such as low socioeconomic status, rural residence, limited awareness of anaemia, and poor access to sanitation and healthcare services contribute to the high burden of anaemia among reproductive-age women and adolescent girls.^(8–10)^

The WHO recommends intermittent iron and folic acid (IFA) supplementation — 60 mg of iron and 2.8 mg of folic acid — for adolescent girls to improve Hb levels, iron status, and reduce anaemia risk. IFA supplementation strategies for anaemia treatment are tailored to the prevalence of anaemia within specific populations.^(11)^ Weekly supplementation is recommended for menstruating girls in areas with 20% anaemia prevalence, and daily supplementation in areas with 40% or higher prevalence.^(1)^ Effective anaemia reduction requires programmes that assess current measures against iron and folate deficiency and address underlying health issues.^(12)^

The Ethiopian government has implemented a range of nutrition interventions aimed at addressing micronutrient deficiencies among adolescent girls. These initiatives focus on enhancing dietary diversity, promoting supplementation, and improving access to fortified foods.^(13)^ As part of the National Nutrition Program II, the government of Ethiopia endorses the provision of weekly iron and folic acid supplementation (WIFAS) for adolescent girls to reduce anaemia. The programme promotes the use of school platforms to ensure that adolescents have access to these essential micronutrients.^(14)^ However, large-scale WIFAS programmes are not being implemented.

Previous studies in low- and middle-income countries have shown that the WIFAS programme significantly reduces anaemia among adolescent girls. In Ethiopia, donor-supported pilot programmes are implementing WIFAS in schools.^(15,16)^ However, its effectiveness is often compromised by various demand- and supply-side barriers at community, school, family, and individual levels. Notably, there is a lack of research documenting the barriers and facilitators of the WIFAS programme implementation among adolescent girls in the country. Therefore, this study aims to explore the demand- and supply-side barriers and facilitators of the WIFAS programme in the Sidama Region of Ethiopia.

## Methods and materials

### Study setting

This study was conducted in ten second-cycle primary schools, two in each of five selected districts implementing the WIFAS programme in the Sidama Region. The region has a population of nearly 5 million and is located 273 kilometres south of Addis Ababa, the capital of Ethiopia. Hawassa City serves as the regional capital. Major crops grown in the region include enset, maize, wheat, barley, and teff, along with fruits, vegetables, and root and tuber crops. Coffee and khat are significant cash crops in the region.^(17)^

Since May 2022, in collaboration with partners, the region has been supplementing weekly iron and folic acid (WIFA) to adolescent girls in nine districts: *Malga, Hawassa Zuria, Shebedino, Dale, Aleta Wondo, Aleta Chuko, Hula, Bona, and Bensa*. The programme is implemented across 112 second-cycle primary schools, benefiting 51,157 girls.^(17)^

### Study design and period

This study followed a qualitative approach to explore the demand- and supply-side barriers and facilitators of the WIFAS programme. Interview and discussion guides were used to facilitate the dialogue among selected groups and individuals from adolescent girls and their mothers, school directors, trained WIFA supplementing teachers, health centre heads, and district health and district education offices and regional health and education bureaus delegates. Data were collected from 1 April to 28 June 2024.

### Sampling procedure

Regional Health Bureau nutrition focal points and Education Bureau WIFAS delegates were contacted for a programme overview across all implementing districts. Five districts (*Aleta Wondo, Bona, Dale, Hula, and Malga*) were randomly selected from the nine WIFAS implementing districts in the region. Two schools were chosen from each of the five selected districts. From each of the selected schools, stakeholders who have significant roles in WIFAS (adolescent girls, trained WIFAS provider teachers, and school principals) were purposely recruited through in consultation with school principals.

Ten focus group discussions (FGD) were held with adolescent girls. Invitation letters were sent to the mothers of the selected girls, inviting them to the school to participate in the study and provide consent for their daughters’ involvement. As a result, ten FGDs with mothers were also conducted. A total of thirty-eight key informant interviews (KIIs) were conducted: two per school (one with a trained WIFAS service provider teacher and one with the school director), one per health centre in each selected district (with the health centre head), two per district offices (one with the health office and one with the education office), and three with regional delegates (one each for the Health Bureau nutrition delegate, Education Bureau WIFAS delegate, and the WIFAS implementing partner). The study involved 217 participants: 38 for KIIs and 179 for FGDs. The profile of the key informant (KI) is summarised and included in the supplementary material.

### Data collection: tool and approach

Survey guides were developed for FGDs and KIIs, addressing both demand- and supply-side barriers and facilitators for the WIFAS programme. Demand-side barriers focused on awareness, cultural beliefs, accessibility, availability, and stigma, while supply-side barriers considered supply chain, training, logistics, stockouts, and quality concerns. Demand-side facilitators included community education, engagement of health workers and leaders, access improvement, and positive testimonials, while supply-side facilitators included supply chain management, trained service providers, community health networks, partnerships, provider training, awareness creation, government support, and monitoring.

Semi-structured interview guides were developed for both the FGDs and the KIIs. The guides were prepared in English following the review of relevant literature^(18–24)^ and then translated into the local language, Sidamu Afoo, by language experts. After receiving 2 d of training, four well-experienced male data collectors with master’s degrees in public health conducted FGDs. Two assistants facilitated each FGD: a moderator who led the discussion, and an assistant who managed recording. All the FGDs and KIIs were conducted in Sidamu Afoo and audio-recorded with the consent of participants. Field notes were concurrently documented in addition to the audio recordings.

The FGDs were conducted in quiet rooms in the schools to avoid distractions until information saturation was reached.^(25)^ This allowed participants to freely express themselves on the issues discussed. Nine to twelve members formed a group for each of the FGDs. All group members were encouraged by the investigator to partake in the discussions.

For the KIIs, the principal investigator contacted and invited the trained WIFAS service provider teachers, school principals, and office experts of different levels to participate. KIIs were conducted in the offices of school principals or in private rooms. The interviews were conducted one on one basis, exclusive to other people to promote privacy. All FGDs and KIIs lasted between 40 and 90 min. The principal investigator was responsible for the conduct of the KIIs.

Following FGDs and interviews, a summary of key issues was shared with participants for validation. Important new issues identified were added to the interview guides for future discussions. Data saturation occurred when participants’ responses to prompts and probes were exhausted.^(25)^

### Data quality assurance

Data quality in this qualitative study was ensured through rigorous methods. Semi-structured interview guides were developed, reviewed, and translated. Experienced data collectors received training, and data collection continued until saturation. All interviews were audio-recorded with participant consent, supplemented by field notes, and summarised for participant validation. Detailed transcriptions were conducted to accurately preserve participants’ expressions. To ensure reliability, data from multiple sources were cross-verified through triangulation. Emerging themes were iteratively refined through subsequent interview rounds.

### Data management and analysis

Data management and analysis were conducted using Open Code software version 4.03. The audio-recorded data were transcribed verbatim by the lead author. A thematic analysis approach, incorporating both inductive and deductive coding, categorised the transcripts into supply barriers, supply facilitators, demand barriers, and demand facilitators. This involved conventional content analysis, using open coding to identify initial concepts, categories, and subcategories, followed by the grouping of similar codes into categories and the subsequent merging of categories into themes aligned with the study objectives. All quoted text reflects the direct words of the study participants translated into English.

#### Rigour and trustworthiness of the study

To ensure the credibility and trustworthiness of the study, a comprehensive methodological framework was implemented. The research employed two primary data collection methods: FGDs and KIIs. These methods were deliberately selected to capture a diverse range of perspectives and experiences related to the IFA supplementation programme, thereby enhancing the depth and richness of the data collected.

The principal investigator conducted a thorough review of existing literature concerning the utilisation of IFA supplementation, as well as the potential barriers and facilitators associated with these supplements. This literature review informed the development of a comprehensive data collection tool that was grounded in established knowledge, ensuring that the study was well-informed and relevant to the context of the supplementation programme.

FGDs were conducted with adolescent girls and their mothers, providing a platform for participants to share their personal experiences and perceptions regarding the IFA supplementation programme. This method facilitated open dialogue, allowing participants to express their views in a supportive environment. The interactive nature of FGDs encouraged participants to build on each other’s responses, which helped to uncover nuanced insights into the barriers and facilitators they encountered. This approach not only enriched the data but also fostered a sense of community among participants, which is crucial for understanding social dynamics related to health interventions.

KIIs were conducted with a diverse group of stakeholders, including school directors, trained WIFAS provider teachers, health centre heads, and nutrition focal points from district health and education offices, as well as regional health and education bureaus. This multi-faceted approach ensured that the study captured a comprehensive view of the barriers and facilitators from both demand and supply perspectives. Engaging with KIs provided valuable insights into institutional perspectives and operational challenges, which are essential for understanding the broader context of the supplementation programme. The combination of FGDs and KIIs allowed for triangulation of data, thereby enhancing the credibility and validity of the findings.

The researcher practised reflexivity throughout the study by reflecting on his biases and beliefs that could influence the research process. This self-awareness enhanced data interpretation, allowing for critical assessment of how personal perspectives might shape analysis and conclusions. Such reflexivity led to a deeper understanding of participants’ experiences and ensured that findings accurately represented their views, free from researcher bias. This approach contributed to a more rigorous and transparent research process, reinforcing the validity of the study’s insights.

### Ethical clearance

This study was conducted in accordance with the principles outlined in the Declaration of Helsinki. Ethical clearance was obtained from the Institutional Review Board (IRB) of the College of Medicine and Health Sciences, Hawassa University ***(Protocol Number-IRB/026/16, Date-12/12/2023)***. Prior to data collection, officials from the regional bureaus, district offices, and school directors were approached to communicate the study’s purposes, and permission was secured. Informed written consent was obtained from each participant in the study, and their information was kept confidential using pseudonymous codes.

## Results

### Socio-demographic characteristics of study participants

This study included 38 KIs and 179 FGD discussants. All interviews were conducted in Sidamu Afoo. KIs’ ages ranged between 24 and 45 years. About two-thirds of the KIs (65.8%) were males, and all of the FGD participants were females. Thirty-four out of 38 KIs and eighty-six out of 179 FGD participants were married. All KIs have a college diploma or higher. Eighteen out of 179 FGD participants did not attend formal education, while the rest attended primary to college education (Table 1).

**Table 1.** Socio-demographic characteristics of key informants (KIs) and focus group (FG) discussants participated in a qualitative study conducted from 1 April to 28 June 2024, in Sidama Region schools, Ethiopia

Socio-demographic characteristics of KIs and FG discussants	Category	Frequency	Percent (%)
Age in year of KIs	24–35	20	52.6
	36–45	18	47.4
Age in year of FG discussants	Mothers (34–56)	86	48
	Adolescent girls (14–19)	93	52
Sex of KIs	Male	25	65.8
	Female	13	34.2
Marital status of KIs	Married	34	89.5
	Single	4	10.5
Marital status of FG discussants	Married (mothers)	86	48
	Single (adolescent girls)	93	52
Education status of KIs	Bachelor of science and above	31	81.6
	College diploma	7	18.4
Education status of FG discussants	No formal education	18	10
	Primary education	109	60.9
	Secondary education	39	21.8
	College diploma and above	13	7.3
Occupation of KIs	Education professionals	26	68.4
	Health professionals	12	31.6
Occupation of FG discussants	Students	86	48
	Housewives	54	30.2
	Merchants	31	17.3
	Employee	8	4.5

^*^Key informants = 38; focus group discussants = 179.

Concerning occupation, KIs from health centres, health offices, and the regional health bureau are health professionals coordinating nutrition activities in their respective offices and supervising WIFAS activities in schools. The school, education office, and education bureau KIs are education professionals responsible for coordinating teaching-learning activities and WIFAS tablet provision in schools (Table 1).

After gathering data through KIIs and FGDs, themes emerged from thematic analysis of qualitative data on programme implementation practices in Sidama region schools: demand- and supply-side barriers and demand- and supply-side facilitators (Table 2).

**Table 2.** A table summarising the themes, sub-themes, and codes that emerged during data management and analysis, with illustrative quotes from interviews and focus group discussions conducted from 1 April to 28 June 2024, in Sidama Region schools, Ethiopia

Themes	Sub-themes	Codes	Meaning of codes
Barriers for utilisation of WIFAS	Awareness of WIFAS tablet	Inadequate awareness	Adolescent girls and their parents were not adequately informed about the tablets. ‘*…the supplementation of the tablets was initiated in schools without adequately informing girls parents about the tablets*’. Mothers’ FGD Participant
		Time constraints	Trained teachers felt they were not given enough time to effectively educate the girls about the tablets. ‘…*We were not given sufficient time to make the girls aware of the tablet*’. IFA Provider Teacher KIs
	Community perception	Misinformation and rumours	The community initially misunderstood the supplements as contraceptives and spread of misinformation within the community. ‘*The community wrongly perceived the supplements as a contraceptive method, believing that the government was providing the tablets in schools to make their daughters sterile*’. School Director KIs
		Shift from resistance to acceptance	Trust in authoritative sources (e.g. teachers, health workers) helped counteract misinformation. ‘*After a HEW and a teacher informed me about the effects of blood loss on girls’ health, including my daughter’s, I encouraged her to take the tablets without missing a dose, and she now takes them properly*’. Mothers’ FGD Participant
	Side effects of IFA tablet	Fear of side effects	Girls reported experiencing side effects. ‘*I didn’t feel well the day I took the tablet; my stomach ached, my appetite decreased, and I had trouble going to the bathroom*’. Girls’ FGD Participant
		Adaptation to medication	Adaptation to side effects and improved adherence. *Over time, the side effects have decreased, and I’ve gotten used to it.* Girl FGD Participant
	Interrupted supplementation	Disruption of Supplementation	Lack of access to tablets during school closures. ‘*After a month of school closure, I face tablet shortages*’. Girls’ FGD Participant
		Teacher and peer support	Teachers and peers support girls to take tablets consistently. ‘*In school settings, teachers and designated facilitator students remind and encourage girls to take their tablets consistently*’. IFA Provider Teacher
		Loss of support systems	During school closures, the absence of peers and teachers support leads to discontinued use of the tablets. ‘*No one reminds or encourages me like teachers and peers do in school during summer*’. Girls’ FGD Participant
Barriers for utilisation of WIFAS	Programme coordination	Role ambiguity	Lack of coordination among stakeholders created confusion. ‘*The lack of well-organized program coordination created confusion among stakeholders regarding roles and responsibilities, leading to overlapping efforts and neglected tasks*’. District Health Office KIs
		Inconsistent service delivery	Uneven service delivery and monitoring compromised effectiveness of the programme. ‘*Inconsistent efforts among the responsible sectors led to uneven service delivery and monitoring, compromising the supplementation of tablets to adolescent girls*’. Regional Health Bureau KI
	Supply chain management	Stockouts	Schools faced stockouts and irregular supply, which interrupted the distribution of IFA supplements to adolescent girls. ‘*The stockouts affected supplement availability and impacted the distribution*’. Regional Health Bureau and District Health Office KIs
		Conflicting attributions of responsibility	Differing perspectives among stakeholders regarding the causes of supply chain problems. ‘*Health centers attributed irregular supply and stockouts at the regional supply branch, while health offices blamed delays in requests and poor supply chain management at the health centers*’. District Health Office and Health Centre KIs
	Targeted coverage	Programme focus	IFA tablets distributed to girls without prior assessment. ‘*The program aimed to prevent anemia by providing tablets to adolescent girls without clinical assessment*’. Regional Partners KIs
	Water scarcity	Hygiene and sanitation	Girls forced to share glasses for taking tablets, increasing the concern of hygiene and disease transmission. ‘*Teachers make us share a glass of water to swallow our tablets. We are worried that this puts us at risk of getting sick from germs*’. Girls’ FGD Participant
		Financial burden	Schools lacking sufficient water forced purchasing water at additional costs. School Director KIs
		Participation	Poor hygiene during distribution could undermine trust in the programme and discourage participation. ‘*Not having enough water in schools not only makes it hard for girls to take their tablets but also undermine trust in the program*’. District Education Office KIs
Facilitators for utilisation of WIFAS	Payment-free IFA tablet	Free access	Free distribution of WIFA tablets in schools improve adherence and effectiveness. ‘*Providing tablets in schools for free is crucial for increasing their use, as it removes financial barriers*’. Regional Partners KIs
	Positive testimonials	Peer influence	Girls who have successfully used IFA tablets serve as trusted peer counsellors. ‘*After hearing about its benefits from a friend who has been taking it, I started taking it*’. Girls’ FGD Participant
		Facilitator girls	Reduced teachers’ workload and improving programme efficiency. ‘*Facilitator girls alert IFA user girls, bring tablets from the storage room, and assist girls in accessing water for swallowing the tablets*’. School Director KIs
	Local leaders support	Kebele leaders	Played role in awareness creation. ‘*Invited women and children’s affairs heads from the kebele delivered a message to girls, and after that, girls started to utilize the tablets*’. IFA Provider Teacher KIs
		Religious leaders	Addressed misconceptions. ‘*Misconception was addressed by involving religious leaders in an awareness creation*’. School Director KIs
	Health extension workers	Build community trust	Disseminate accurate information. ‘*HEWs dispelled myths and spread vital information about the tablets, ensuring adherence to the supplements*’. Health Centre KIs
	Training	Counselling skill	Helped develop knowledge and skills. ‘*The training I received helped me how to approach the girls and counsel them about the tablets use*’. IFA Provider Teacher KIs
		Programme fidelity	Training improved programme effectiveness. ‘*Training and regular performance reviews have significantly improved program implementation over time*’. District Health Office KIs
	Supervision	Problem identification	Helped adjust strategy. ‘*It helped school directors and IFA provider teachers identify problems, adjust strategies, and adhere to protocols*’. School Director KIs
		Technical support	Improved tablet providers’ commitment. ‘*Timely supervision really helped make the program work well and improved the quality of services provided*’. Regional Education Bureau KI
		Monitoring and evaluation	Ensure consistent and quality service delivery. ‘*Regular quarterly review meetings improved the program’s implementation*’. District Education Office KIs

WIFAS, weekly iron and folic acid supplementation; IFA, iron and folic acid; FGD, focus group discussion; KI, key informant.

### Barriers for utilisation of WIFAS among adolescent girls

#### Awareness for IFA tablet

Prior to implementing the WIFAS programme in the schools, district health and education office nutrition focal points and school directors, in coordination with health extension workers (HEWs), conducted campaigns to raise community awareness about the programme. In schools, trained IFA provider teachers and health workers from nearby health centres were tasked with delivering health and nutrition education to adolescent girls. However, as school KIs explained, the nutrition education provided to adolescent girls in school lacked sufficient time to raise awareness about the tablets for the girls. An IFA provider’s teacher from one school said the following during the interview: ‘…*the process was hasty; when we started the service after we got back from the training, we were not given sufficient time to make the girls aware of the tablet*’.

According to the health and education office focal points, the awareness creation campaigns conducted in the community were non-specific and did not yield the intended results. They emphasised that the supplementation of the tablets was initiated in schools without adequately informing parents about the tablets (benefits and potential risks) being given to their daughters. FGD discussants (mothers of the girls) reported that they were not adequately informed about the benefits and potential risks of the tablets given to their daughters. As a result, they sought information from neighbourhood women whose daughters attended the same school and directly questioned the teachers. One of the mothers’ FGD attendants stated, ‘…*When my daughter started taking the medication, no one informed me about it. As a result, I sought information from a woman living next door, whose daughter attended the same school. She had visited the school to ask the teachers about the medication given to her daughter*’.

Similarly, FGD participants (adolescent girls) explained that at the beginning of the supplementation of the tablets in their school, they did not fully understand the benefits of IFA tablets for health, particularly in preventing anaemia and supporting proper growth. As a result, they were less likely to seek or accept these supplements.

### Community perception

As KIs and mother FGD participants indicated, at the beginning of the programme, the community wrongly perceived the supplements as a contraceptive method, believing that the government was providing the tablets in schools to make their daughters sterile. However, after frequent awareness creation campaigns, they understood that the tablets were given to their daughters to prevent anaemia. One of the FGD discussants mentioned that after a teacher explained how blood loss affects girls’ health at her daughter’s age, she began to support her daughter in taking the tablets provided at school to prevent anaemia. This concern was echoed by another participant who shared a similar experience:

…Even though I initially discouraged my daughter from taking the tablets given at school after hearing women in my neighbourhood claim that the government intended to make their daughters sterile, after a health extension worker informed me about the effects of anemia on girls like my daughter and the purpose of the tablets, I encouraged her to take tablets without missing a single dose, and she is now taking them properly.

### Side effects of IFA tablets

Girls in the FGDs said that the side effects of IFA supplements affected how often they took the tablets, making it harder for them to stick to a routine. The main issues they faced were stomach pain and nausea. They mentioned that these side effects were worse when they took the tablets on an empty stomach in the morning shift. One of the discussants complained,

…I didn’t feel well the day I took the tablet; my stomach ached, my appetite decreased, and I had trouble going to the bathroom. Because of this, I would have liked to stay out of school on the day the medicine was given, but I came because my parents wouldn’t let me miss school; over time, the side effects have decreased, and I’ve gotten used to it.

One of the teachers who provides IFA voiced a supportive reflection, ‘…*when we first started this service, we were worried about girls missing school on the day they took the tablet; but now, that issue is resolved*’.

#### Interruption of the supplementation due to school closures

KIs reported that school closures during the summer (Kiremet) pose a significant challenge to the continuous supplementation of tablets for adolescent girls, potentially undermining efforts to prevent iron deficiency and anaemia. Since schools serve as the primary distribution points for these tablets, their closure disrupts regular access, leading to missed doses and reduced compliance. KIs also underscored that school closures are particularly concerning for adolescent girls in rural areas, where community-based supplementation programmes are non-existent or alternative healthcare facilities providing the tablets are absent.

Trained WIFAS tablet provider teachers noted that beyond physical access, school closures also remove essential peer and teacher support systems crucial for reinforcing adherence to supplementation. They emphasised that in school settings, teachers and designated facilitator students remind and encourage girls to take their tablets consistently, providing motivation and addressing concerns about side effects. FGD discussant girls also acknowledged that school closures impact tablet utilisation, stating that the absence of encouragement and tablet shortages during the summer led them to discontinue use. One discussant shared, ‘*…School closures during summer disrupt my regular tablet use; no one reminds or encourages me like teachers and peers do in school. After a month, I also face tablet shortages*’. However, KIs from *Malga* and *Hula* district education offices disputed the issue of shortages, stating that girls were provided with enough tablets to take home for summer use.

### Programme coordination

According to KIs, the lack of well-organised programme coordination among responsible sectors was a barrier to WIFAS in the study area. This situation created confusion among stakeholders regarding roles and responsibilities, leading to overlapping efforts and neglected tasks. Sometimes, the lack of clarity resulted in communication gaps that delayed decision-making and disrupted the flow of information. Inconsistent efforts among the responsible sectors led to uneven service delivery and monitoring, compromising the supplementation of tablets to adolescent girls. Poor programme organisation also impacted the reporting system of the programme, as an IFA provider teacher explained.

### Supply chain management

Regional and district nutrition officers mentioned that health centres receive their IFA supplies directly from the regional branch of the Ethiopian Pharmaceuticals Supply Service. Health centre pharmacists then distribute the tablets to schools participating in the WIFAS programme. These officers oversaw tablet delivery to schools and delivery to the girls. During supervisory visits, they ensured that remote and hard-to-access schools received supplies.

In the study area, the distribution of IFA supplements to schools encountered stockout challenges. KIIs from schools highlighted that the stockouts they faced at one time affected supplement availability and impacted the distribution of IFA supplements to adolescent girls in schools. This inconsistency faced for a month so far has led to interruptions in distribution, causing gaps in the girls’ supplementation schedules and making it difficult for them to receive their required doses regularly. According to IFA provider teachers, apart from the irregular supply for a month, the shortage of tablets was not their main concern; instead, they emphasised that health centres provided tablets close to their expiration date, impacting the quality of services offered.

The KIIs from health centres expressed concerns about the root of the problem, attributing the irregular and close-to-expiration tablet distribution to the regional branch of the Ethiopian Pharmaceuticals Supply Service. However, KIIs from health offices disagreed with this view, indicating that the irregular tablet supply to schools stemmed from poor supply chain management at the centres, resulting from delays in requesting tablets promptly from the regional branch of the Ethiopian Pharmaceuticals Supply Service.

#### Target coverage

KIs and participants in the adolescent girls’ FGD were asked about the diagnostic services provided to tablet utilisers before starting the service to assess nutrient levels such as iron (Hb) and folate. They were also asked if the same nutrient levels were checked after a specified time to evaluate the tablets’ effect on these nutrients. KIs stated that the programme was targeted at anaemia prevention, providing tablets to adolescent girls without assessing their clinical condition or nutrient levels.

However, FGD participant girls complained,

…We started taking the tablet at school without knowing whether we had anemia or not; we have been taking the tablet continuously for more than two years; they tell us to take it, but they do not tell us the changes the tablet we took has made in our health; it is tiring to just keep taking it.

School directors were also asked why they weren’t inviting health professionals from the nearby health centre to conduct anaemia screenings and Hb tests for the girls taking the tablets. They explained that Hb testing is not pursued because it incurs fees. They mentioned that when they ask health centre professionals to do symptom-based screenings, the professionals refuse, stating they only do them if invited by partners.

Teachers who provide IFA often mentioned that they encountered delays in receiving timely professional support from health centres when needed. During a KII, a teacher who offers IFA supplements shared this issue: ‘…*We requested professional support from a nearby health centre when a girl experienced unusually heavy menstrual bleeding after starting to take the tablets; unfortunately, they didn’t respond until a week later*’.

### Water scarcity

Water scarcity was one of the major barriers in the study area that challenged the supplementation of WIFA. School KIs said that water is essential for taking the tablets and keeping things clean during distribution. However, because there isn’t enough water at school, students have trouble taking these supplements regularly, which makes it harder for them to stick to the programme.

‘*There is no water in our school, and we make the girls bring water from home on the day they receive the tablet; but most of the time, we buy a 20-liter jar of water ourselves for 50 birr (0.41 USD), so the program made us incur additional expenses*’, complains a 35-year-old school director from *Malga* district, *Wojigra* school.

Similarly, adolescent girls are worried that not having enough water in schools not only makes it hard for them to take their tablets but also leads to unhygienic conditions when the supplements are given out, which could increase the chances of spreading diseases. FGD discussant girls complained, ‘…*because there is no enough water, teachers make us share a glass of water to swallow our tablets. We are worried that this puts us at risk of getting sick from germs*’.

### Facilitators for utilisation of WIFAS among adolescent girls

#### Payment-free IFA tablet

Most KIs emphasised that providing tablets in schools for free is crucial for increasing their use, as it removes financial barriers. They noted that having WIFA supplements available at no cost makes it easier for students to take them regularly. With easy access in schools, students are more likely to follow the supplementation schedule, improving the programme’s effectiveness. A mother in the discussion also added, ‘…*Without free tablets, many girls’ parents couldn’t afford them. Giving them for free let’s all girls access them equally, whether rich or poor. She said the organization behind this deserves thanks*’.

#### Positive testimonials/success cases

FGD discussant girls acknowledged that positive exemplars/success cases, acting as councillors, helped raise awareness among new service users because these girls trust their peers more than teachers, thus improving utilisation and compliance. One of the discussants explained,

…Before I started taking the tablet, my attitude about it was not good. I couldn’t believe it when my teacher recommended it to me. But after hearing about its benefits from a friend who has been taking it, I started taking it. Now, I feel better — no more fatigue, headaches, or dizziness during my period. I’m now teaching other girls about its benefits with my friends.

Similarly, the participation of facilitator girls in the distribution of tablets streamlined the programme in the school. According to school directors, facilitator girls were selected from tablet user girls based on their long-term compliance with supplementation and trained to support the tablet provider teachers. They alert IFA user girls a day before supplementation, bring tablets from the storage room to the supplementation site, and assist girls in accessing water for swallowing the tablets.

A 28-year-old IFA provider teacher from Hula district elaborated on the roles of the facilitator girls as follows:

How difficult would the work be for teachers if the facilitator girls did not participate in the distribution of WIFAS tablets? How could we alert all the user girls from their respective classrooms? How could we arrange water for swallowing the tablets? How could we line up the girls in a row to give out WIFAS tablets? Their presence has reduced all this workload for us; they deserve respect.

#### Local leaders support

Involvement of local leaders in awareness creation could reveal that robust community support plays a pivotal role in enhancing girls’ utilisation of supplements. Most of the school KIs mentioned that the involvement of the kebele leaders improved the utilisation of tablets at their school. One trained WIFAS provider said the following during the interview:

…Our school director invited women and children’s affairs heads from the kebele leaders on the day of tablet supplementation to deliver a message to girls about the importance of tablet use in school, and after that, girls started to utilize the tablets properly.

Similarly, the involvement of local religious organisation leaders in promoting supplementation awareness and the presence of supportive social networks positively influence tablet utilisation. A director from one school explained the following during a KII:

…We faced a challenge at the beginning of WIFA supplementation where girls perceived the tablets as drugs given to prevent fertility. This misconception was addressed by involving religious leaders from the kebele in an awareness creation session where the leaders taught them that WIFAS is provided to prevent anemia.

#### Health extension workers

Engaging HEWs in community awareness significantly improved IFA supplementation for adolescent girls in schools. At the beginning of the programme implementation, HEWs spread vital information about these supplements, dispelled myths, and highlighted their health benefits. School directors noted HEWs’ vital role in awareness campaigns that enhanced community understanding and acceptance of the programme. HEWs’ involvement, as explained by IFA teachers, raised trust among girls, ensuring continued participation and adherence to the supplementation plan.

#### Training

KIs were asked about the importance of providing training to IFA provider teachers in terms of equipping them with the necessary knowledge of programme guidelines, dosing, administration techniques, side effect monitoring, and addressing common misconceptions, enhancing their skills in counselling girls and parents, promoting adherence, and addressing potential participation barriers. All KIs acknowledged training as beneficial for providers to enhance skills, foster confidence, and ensure programme fidelity for positive health outcomes.

One of the IFA provider teachers explained the following during KII:

…the training I received helped me understand anemia and its consequences; it taught me how to approach the girls and counsel them about nutrition, how to handle the tablet and give it to the girls, and, most importantly, as a father of two adolescent girls, it supported me in improving the nutrition of my daughters at home.

A KI from the health office of one district mentioned that with the support of a partner organisation, the basic training given for new IFA provider teachers and the regular performance reviews every 3 months have improved the IFA programme in our district significantly in the last 3 years.

## Supervision

KIs from regional and district offices reported that timely supervision really helped make the programme work well. They also explained that timely oversight helped improve the quality of services provided and the commitment of service providers. KIs at the school level also agreed, saying that the technical support they received made sure girls got the supplements on time and in the right way. During FGDs, adolescent girls also mentioned that ‘…supervisors from health and education offices often came to their schools, asked them how they used the tablets and watched how the tablets were given out at the schools’.

In addition, regular quarterly review meetings organised by partners improved the programme’s implementation in schools. School directors noted these meetings helped them spot problems, adjust strategies, and stick to protocols, ensuring timely and quality delivery of supplements. IFA provider teachers also mentioned that the meetings helped them track progress, share best practices, and stay committed, leading to consistent supplementation of tablets for adolescent girls.

## Discussion

Our study explored the demand- and supply-side barriers and facilitators of the WIFAS programme in the Sidama Region. Low awareness of IFA tablets, incorrect community perceptions of supplements, fear of side effects, interrupted supplementation due to school closures, inadequate professional support, poor programme coordination, ineffective supply chain management, and water scarcity were identified as barriers to programme implementation in the study area. On the other hand, the provision of tablets free of charge, the use of successful cases for counselling, the involvement of facilitator girls, and the participation of HEWs in awareness campaigns, support from local leaders, training, and regular supervision were identified as facilitators of programme implementation in the study area.

According to this study, low awareness among adolescent girls regarding IFA supplementation, along with the misconception held by the community that the tablets provided in schools would make their daughters sterile, challenged the implementation of the programme. This finding is consistent with a study conducted in southern Ethiopia,^(20)^ which highlights that inadequate awareness about IFA and its benefits often leads to misconceptions that can hinder adherence to supplementation programmes. When parents are not adequately informed about the health benefits of IFA, they may develop negative perceptions that discourage their daughters from participating in supplementation.^(26)^ Studies have also shown that cultural beliefs and misinformation about IFA tablets create barriers to acceptance and adherence, especially in communities with low educational attainment and strong traditional beliefs.^(20,27)^

The study found that fear of side effects from IFA supplements hindered tablet use among adolescent girls. Adverse effects can also reduce adherence to supplementation programmes. In line with this, a Ghanaian study revealed that 27% of adolescent girls experienced undesirable changes, such as heavy menstrual flow, impacting their willingness to consume IFA tablets.^(18)^ Similarly, girls in Botswana voiced concerns about side effects, leading to reduced uptake.^(28)^ This may be due to the fact that girls were not given comprehensive health education that addresses potential side effects and promotes the benefits of IFA supplementation.

This study presented that school closures disrupt adolescent girls’ access to WIFA, leading to missed doses and reduced compliance. Consistent with prior research, schools serve as vital distribution hubs, and closures create tablets shortage.^(29)^ The absence of peer and teacher support further weakens adherence, reinforcing findings that social networks enhance compliance.^(15)^ Tablet shortages, especially in summer, exacerbate discontinuation, aligning with studies on supply chain instability.^(26)^ These findings highlight the need for alternative distribution strategies and stronger community support systems.

The current study further notes absence of targeted coverage significantly impacts the WIFAS programme. The programme effectiveness and satisfaction of the service users cannot be ensured without timely assessing the blood level of nutrients. This results in lower adherence to supplementation programmes, as students may not understand their personal health risks or the importance of IFA intake. A study conducted in southern Ethiopia showed that the lack of targeted assessment negatively affects the consumption of IFA supplements among girls.^(20)^ Furthermore, the inability to monitor and evaluate the effectiveness of supplementation programmes can perpetuate the cycle of anaemia, as schools may not adjust their strategies based on the actual health outcomes of their students.^(27)^

The current study revealed a gap in programme coordination among sectors implementing the WIFAS programme in schools, leading to confusion over roles and responsibilities. According to KIs, this poor organisation hampered communication, resulting in misunderstandings about programme needs and delays. They also underlined that such misalignment can disrupt demand forecasting and resource allocation, causing stockouts or excess IFA tablets nearing expiration. Finally, strained relationships among stakeholders in district offices contributed to poor supply chain management for a month.

The study found that schools with the WIFAS programme do not have regular access to safe drinking water. As all school KIs reported, schools have no water source on the premises. Due to this, adolescent girls are obliged to fetch water by travelling long distances or bringing water from home on the days they receive the tablets, or school directors must buy water, incurring additional expenses. FGD discussant girls noted that the water shortage hindered not only the swallowing of tablets but also negatively affected hygiene practices necessary for their safe consumption. A study conducted in Ghana indicated that the unavailability of water in schools was an obstacle for adolescent girls to ingest the tablets.^(30)^ In addition, another study conducted in Indonesia identified the scarcity of water in schools as a challenge in implementing the WIFAS programme.^(31)^ According to the study, girls go out of the school compound in search of water to swallow the tablets, wasting their valuable time.

Among the facilitators of WIFAS programme, first and foremost, the study found that supplementing IFA tablets at no cost ensures equal access for all adolescent girls, regardless of socioeconomic status, thereby addressing a key barrier to health equity. This finding aligns with previous research in Ghana, which demonstrated that free distribution of IFA tablets in low-income communities led to a marked increase in uptake among girls.^(32)^ Similarly, a study conducted in southern Ethiopia highlighted that financial barriers are a primary hindrance to accessing essential nutrition services, underscoring the importance of cost-free interventions in promoting equitable health outcomes.^(20)^ Providing IFA tablets to adolescent girls without payment not only facilitates greater adherence but also fosters a more inclusive approach to adolescent health interventions.^(33)^

Second, as part of their experience with tablet use, adolescent girls reported that use of positive exemplars as peer counsellors increased awareness and trust among new users, leading to better tablet use and compliance. School informants noted that facilitator girls, chosen for their long-term adherence to supplementation, significantly improved the programme’s efficiency. They helped by informing users about tablet distribution, managing logistics, and providing direct support. A study in Ghana showed that counselling girls by peer educators, who were motivated by the positive results of using the tablets, improved the utilisation of tablets among girls.^(32)^ According to this study, using peer counsellors created a supportive environment and improved the programme’s outcomes for adolescent girls.

Third, involving local leaders in raising awareness significantly enhances girls’ utilisation of supplements, as evidenced by insights from school KIs. Many noted that Kebele leaders played a crucial role in improving tablet usage. For instance, a trained IFA provider highlighted that the school director invited local women and children’s affairs heads to communicate the importance of tablet use, which led to increased compliance among girls. Similarly, the participation of religious leaders in awareness sessions helped dispel misconceptions, such as the belief that the tablets were fertility drugs. This finding is in line with a study conducted in Hadiya Zone, Ethiopia.

Fourth, as KIs noted, training provided to IFA provider teachers enhanced their understanding of programme guidelines, dosing, administration techniques, and side effect monitoring. The training also improved their counselling skills for girls and parents and helped address barriers. This ensured programme fidelity. In line with this, a study conducted in southern Ethiopia emphasised the importance of training, indicating that a lack of trained nutrition service providers in schools might lead to low utilisation of the services among adolescent girls.^(20)^ Another study in Ghana also reported that providing training is a cost-effective method that, if applied properly, can improve programme fidelity and strengthen the behaviour change component.^(32)^

Moreover, the importance of timely supervision was underscored by KIs from regional and district offices, who reported that regular oversight improved service quality and provider commitment. School-level informants corroborated this, noting that technical support ensured that girls received supplements correctly and on time. Adolescent girls also acknowledged the presence of supervisors from health and education offices, indicating that such oversight was crucial for proper tablet administration. Similar to our findings, a study conducted in India reported that regularly supervising the supplementation of IFA to adolescent girls significantly improved the implementation of the programme in schools.^(34)^ Complementing supervision with review meetings provided a platform for school directors and IFA provider teachers to identify challenges, adapt strategies, and share best practices.

### Strength and limitation of the study

The study utilised a mixed-methods approach, incorporating KII and FGDs to gather diverse perspectives from various stakeholders. The triangulation of perspectives enriches the depth and reliability of the study findings. However, it lacked input from national partners in the WIFAS initiative, including the Ministries of Education and Health, as well as international partners. Despite efforts to minimise biases, factors such as recall and social desirability may still have influenced the results.

## Conclusion

This study highlights several critical barriers that hinder the effective implementation of Iron and Folic Acid (IFA) supplementation programmes. Key challenges include low awareness of IFA tablets, negative community perceptions, and concerns about potential side effects and interruption of supplementation. Moreover, structural limitations such as the lack of targeting, poor programme coordination, inadequate supply chain management, and water scarcity further impede programme success.

Conversely, the study identified key facilitators that can enhance programme effectiveness. Providing free IFA tablets, leveraging positive testimonials from beneficiaries, securing support from local leaders, offering comprehensive training for service providers, and ensuring regular supervision are essential strategies to improve programme uptake and sustainability.

## Recommendations

To enhance the effectiveness of the IFA tablet supplementation programme, targeted awareness campaigns should be developed to educate the community on the benefits of IFA tablets while addressing prevalent misconceptions and fears. Engaging local leaders and influencers is crucial for fostering positive perceptions, as their support can help dispel myths and encourage greater participation in the programme.

Training and capacity building for service providers are essential to ensure they are adequately equipped to administer the programme and address community concerns. Regular training sessions will enhance their skills and knowledge, leading to more effective programme delivery. Moreover, improving supply chain management is necessary to maintain a consistent and adequate supply of IFA tablets, reducing the risk of shortages that could deter participation.

The WIFAS programme requires inputs from both the educational and health sectors. Both sectors should establish a robust monitoring and evaluation framework to conduct ongoing assessments of programme effectiveness and to facilitate the collection of feedback from participants, enabling data-driven adjustments. Finally, concerning water scarcity, all actors in the supply chain should collaborate and develop strategies to mitigate these issues, ensuring the programme’s success in the schools.
